# A combined approach of DNA metabarcoding collectively enhances the detection efficiency of medicinal plants in single and polyherbal formulations

**DOI:** 10.3389/fpls.2023.1169984

**Published:** 2023-05-15

**Authors:** Tasnim Travadi, Abhi P. Shah, Ramesh Pandit, Sonal Sharma, Chaitanya Joshi, Madhvi Joshi

**Affiliations:** Gujarat Biotechnology Research Centre (GBRC), Department of Science and Technology, Government of Gujarat, Gandhinagar, Gujarat, India

**Keywords:** authentication, DNA metabarcoding, herbal medicines, next generation sequencing, pharmacovigilance

## Abstract

**Introduction:**

Empirical research has refined traditional herbal medicinal systems. The traditional market is expanding globally, but inadequate regulatory guidelines, taxonomic knowledge, and resources are causing herbal product adulteration. With the widespread adoption of barcoding and next-generation sequencing, metabarcoding is emerging as a potential tool for detecting labeled and unlabeled plant species in herbal products.

**Methods:**

This study validated newly designed *rbcL* and *ITS2* metabarcode primers for metabarcoding using in-house mock controls of medicinal plant gDNA pools and biomass pools. The applicability of the multi-barcode sequencing approach was evaluated on 17 single drugs and 15 polyherbal formulations procured from the Indian market.

**Results:**

The *rbcL* metabarcode demonstrated 86.7% and 71.7% detection efficiencies in gDNA plant pools and biomass mock controls, respectively, while the *ITS2* metabarcode demonstrated 82.2% and 69.4%. In the gDNA plant pool and biomass pool mock controls, the cumulative detection efficiency increased by 100% and 90%, respectively. A 79% cumulative detection efficiency of both metabarcodes was observed in single drugs, while 76.3% was observed in polyherbal formulations. An average fidelity of 83.6% was observed for targeted plant species present within mock controls and in herbal formulations.

**Discussion:**

In the present study, we achieved increasing cumulative detection efficiency by combining the high universality of the *rbcL* locus with the high-resolution power of the *ITS2* locus in medicinal plants, which shows applicability of multilocus strategies in metabarcoding as a potential tool for the Pharmacovigilance of labeled and unlabeled plant species in herbal formulations.

## 1 Introduction

The herbal commodity market is thriving, owing to the widespread belief that traditional medicine is natural and thus safer, thereby promoting good health and sustainable life policies. But as the market expands, the shortage of genuine resources and lack of taxonomic knowledge challenge the authenticity of herbal drugs and increase the incidence of economically motivated or unintentional adulterations and/or substitutions (Raclariu et al., 2018a). Strict pharmacovigilance is necessary to retain the trust and safety of consumers and their health. However, the regulatory guidelines for medicinal plants often blur the line between foods and therapeutics and vary from nation to nation. To close this research gap, regulatory bodies must implement more reliable, universal, and robust detection methods (Shetti et al., 2011).

Nowadays, various pharmacopeia advocate DNA-based methods such as DNA barcoding and species-specific PCR assay to authenticate herbal raw material. DNA is more stable, unaffected by external factors, and invariably present in almost all plant tissues (Wu and Shaw, 2022). In addition, the DNA-based results are independent of seasonal variations in the age of the plant, which in the case of chemical marker-based methods vary significantly (Parveen et al., 2016). Therefore, the results of DNA-based methods are free from subjectivity, accurate, and provide a universally accepted platform for the authentication of botanicals in a wide range of food and herbal products (Lo and Shaw, 2019). The advent of DNA barcoding is the first step in this direction, as barcoding gives resolution up to species level (Hebert et al., 2003). There are 17 potential barcode regions (*matK, rbcL, ITS, ITS2, psbA-trnH, atpF-atpH, ycf5, psbKI, psbM-trnD, coxI, nad1, trnL-F, rpoB, rpoC1, atpF-atpH*, and *rps16*) for plants, having different degrees of universality, specificity, and taxa resolution power that extensively used in the authentication and identification of medicinal plants (Parveen et al., 2016; Kress, 2017). However, DNA barcoding (Vassou et al., 2016) and species-specific assays (Sharma and Shrivastava, 2016; Travadi et al., 2022a; Travadi et al., 2022b) cannot resolve presence of multiple plant species in a single sample (Mishra et al., 2016), that limitation could be overcome by DNA metabarcoding.

DNA metabarcoding combined the strengths of next-generation sequencing and barcoding for detecting multiple taxa in samples (Coghlan et al., 2012). Using a single plant barcode for species-level identification has proven challenging due to the great diversity, relatively slow molecular evolution, frequent cross-pollinations, and hybridization in the plant kingdom; henceforth, different barcodes show different degrees of taxon specificity (Fazekas et al., 2009). To precisely identify the plant species in the sample, multi-barcode approaches have become more prevalent. Xin et al. (2018) and Frigerio et al. (2021) employed a multi-barcode approach of *ITS2* and *trnL* for various Chinese medicine and herbal teas. However, these studies also highlighted the limitations of DNA metabarcoding applications for authentication of herbal products due to variability in degrees of universality and resolution power of barcodes for specific taxa, a lack of a curated database, and a robust bioinformatics pipeline. To overcome these constraints, there is a need for screening of new barcodes and new variable regions within the same barcode for authentication of the herbal products.

Therefore, the aims of the present study were: 1) to develop a new *rbcL* and *ITS* metabarcode for the detection of medicinal plant species, 2) to validate the primers specificity and efficiency using mock controls and 3) to see whether a multi-barcoding approach could be used for the pharmacovigilance of the herbal products? (17 different single plant formulation and 15 polyherbal market formulations in this study), and 3) to see whether a multi-barcoding approach could detect targeted plant species in herbal formulations?

## 2 Materials and methods

### 2.1 Collection of reference plant material and herbal products

Reference plant materials were collected with the aid of a taxonomist from the Maharaja Sayajirao University (MSU), Vadodara (Gujarat, India) and the Directorate of Medicinal and Aromatic Plants Research (DMAPR), Anand (Gujarat, India). As described earlier, reference plant materials were authenticated by Sanger sequencing of *rbcL* gene (Pandit et al., 2021), and sequences were submitted to the NCBI database (accession number MW628906 to MW628936). Voucher specimens were developed and deposited in our institutional herbarium.

32 herbal products were collected by blind sampling from the local market and e-commerce, with 17 single drugs and 15 polyherbal formulations. Single drugs include four Tulsi (*Ocimum tenuiflorum*) powders, five Gokhru (*Tribulus terrestris*) powders, three Shatavari (*Asparagus racemosus*) powders, two Vasa (*Justicia adhatoda*) products, and one each of Bhringraj (*Eclipta alba*), Ashwagandha (*Withania somnifera*), and Arjuna (*Terminalia arjuna*) powder. Polyherbal formulations include three market samples of Trikatu (has three plant species), three samples of Sitopladi [comprises five constituents, only three of which are plant species; the other two are sugar and Vanshlochan (the female bamboo exudate), hence these two constituents were not considered while analyzing the data expecting absence of DNA for these two], four samples of Rasayana (has three plant species), four samples of Hingwashtak (has seven plant species), and one sample of Talisadi [comprises eight constituents, only six of which are plant species; the other two are sugar and Vanshlochan (the female bamboo exudate), hence these two constituents were not considered while analyzing the data expecting absence of DNA for these two].

### 2.2 Primer designing

To design the metabarcodes for *ITS2* gene, *ITS2* sequences of Magnoliophyta from the BOLD database were downloaded and curated, particularly for the length. For the *rbcL* gene, we used 1,776 sequences of our *in-house* sequencing project submitted to the BOLD database. To design universal barcodes, *rbcL* gene sequences were filtered by length between 450-600 bp. At the end, 1,465 and 1,701 *ITS2* and *rbcL* sequences were retained to design the metabarcode. These sequences were preceded for multiple sequence alignment separately (*ITS2* and *rbcL*) using BioEdit 7.2. HYDEN (HighlY DEgeNerate primers) software (Linhart and Shamir, 2005) to design degenerate primers, where the maximum of 3 degeneracy per primer was allowed. The designed primers were checked for amplicon length using NCBI primer BLAST (Altschul et al., 1990). *rbcL* reverse primer sequence was designed in this study, while forward primer sequence was obtained from Maloukh et al. (2017). To synthesize fusion primers, forward primers of *rbcL* and *ITS2* were tagged with the Ion torrent adapter and a ten bp multiplex identifier barcode. In contrast, reverse primers were tagged with the P1 adapter. Nucleotide sequence of the designed primers and their amplicon length are shown in **Table 1**.

**Table 1:** Primer sequences of *rbcL* and *ITS2* metabarcode with their annealing temperature.

Metabarcode	Primer	Sequence (5’ → 3’)	References	Annealing Temperature	Amplicon Length
*rbcL*	*rbcL-F*	ATGTCACCACAAACAGAGACTAAAGC	20	60°C	320-350 bp
	*rbcL*-R	GTARCVRAMCCTTCTTCAAAAAGGTC	This study		
*ITS2*	*ITS2*-F	CRRAATCCCGTGAACCATCGAGTCYT	This study	60°C	310-330 bp
	*ITS2*-R	AGCGGGTRRTCCCRCCTGACYTG	This study		

### 2.3 PCR Optimization and library preparation

The library preparation process became a single-step process with barcoded fusion primers. The PCR optimization with each barcoded fusion primer was done with 45 different plant DNA listed in **Supplementary Information Table S1**. Thermal cycler conditions, especially primer annealing temperature, were optimized for *rbcL* and *ITS2* primer pairs with the following conditions. PCR mixture containing 10 µL Emerald Master mix (2X) (TaKaRa), 2 µL total genomic DNA (10-15 ng/µL), 1 µL of forward (5 pmol), 1 µL of reverse primer (5 pmol), 1 µL BSA (2 mg/mL) and 5 µL PCR grade water with the following thermal cycling conditions. Initial denaturation 95°C for 5 minutes, followed by 30 cycles of 95°C for 1 minute, for primer annealing a temperature gradient of 50°C to 60°C with an interval of 2°C for 30 seconds and 72°C for 1 minute, and final extension 72°C for 5 minutes. 2.4 Preparation of different mock controls

**Figure 1:**
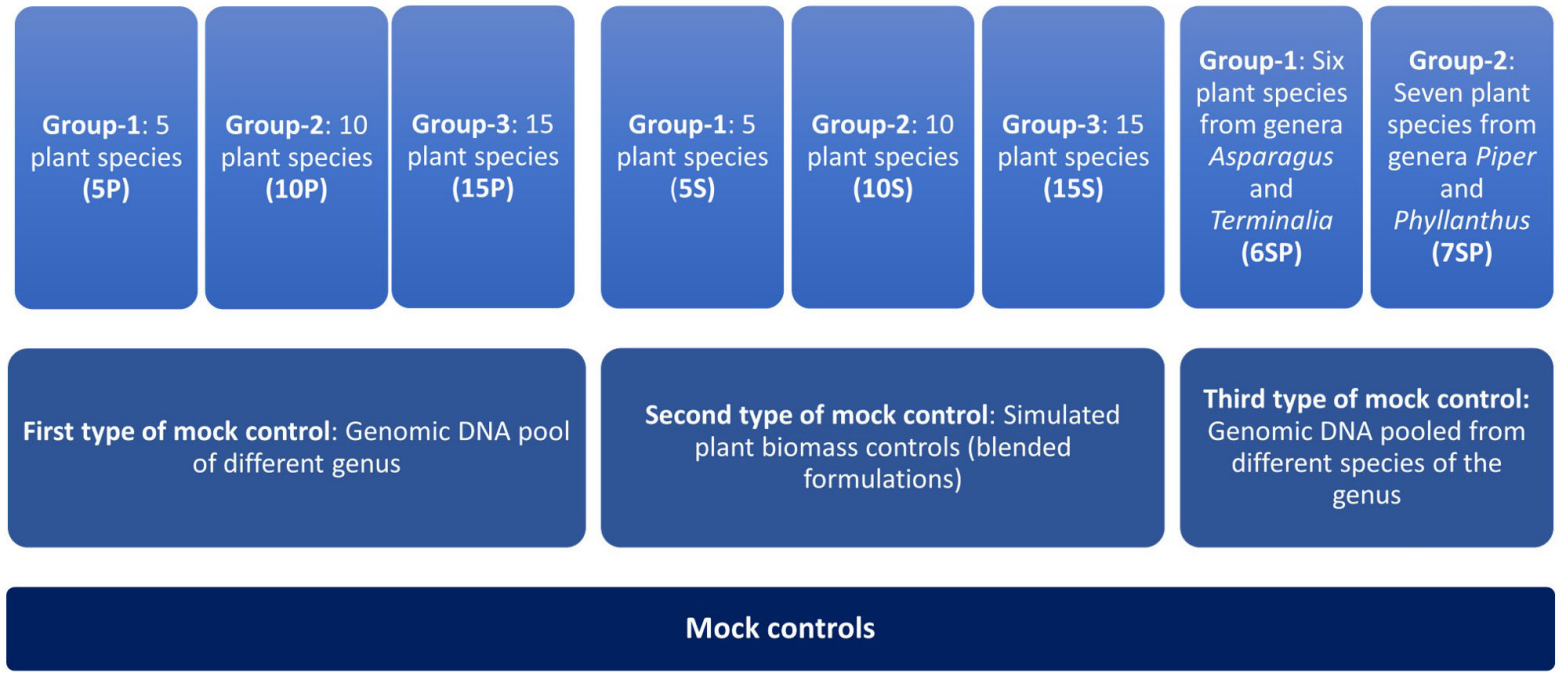
Schematic representation of different types of mock controls prepared in this study. First and second type of mock controls has 3 different groups comprising 5, 10, and 15 plant species. Third type of mock control has 2 different groups, one with a gDNA pool of six plant species from genera *Asparagus* and *Terminalia* and another with a gDNA pool of seven plant species from genera *Piper* and *Phyllanthus*.

**Figure 2:**
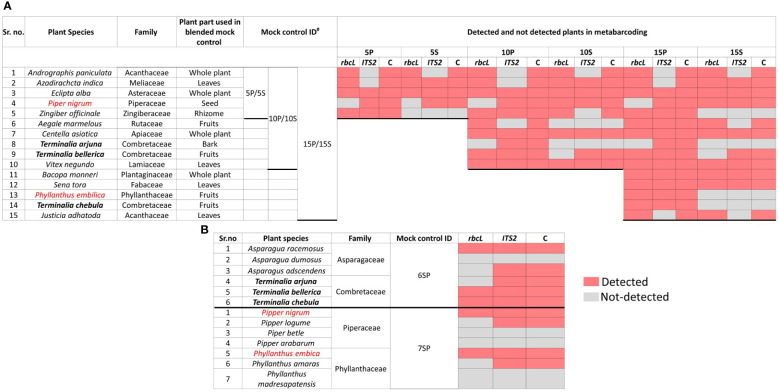
The distribution of predefined herbal species detected in each mock control with *rbcL*, *ITS2*, and combined metabarcoding approach. **(A)** Detected and undetected plant species with *rbcL*, *ITS2*, and combined approach in type one mock control, i.e., genomic DNA pool of different genera, and type two mock control, i.e., simulated plant biomass control (blended formulations) comprising three groups having five plant species (5P/5S), ten plant species (10P/10S) and 15 plant species (15P/15S). **(B)** Detected and undetected plant species with *rbcL*, *ITS2*, and combined approach in a third type of mock controls (i.e., genomic DNA pooled from different species of the genus) comprising two different groups, one having six plant species (6SP) from genera *Asparagus* and *Terminalia* and another having seven plant species (7SP) from genera *Piper* and *Phyllanthus*. Detected plant species are represented in pink, while undetected plant species are represented in grey. Plant species, their family, plant parts used in type two mock controls (simulated plant biomass controls or blended formulations), and mock control IDs are also indicated in the figure. P: gDNA plant pool controls, S: simulated blended plant pool (biomass control), C: a combined approach.

Three different types of controls were prepared as follows: Control 1) genomic DNA from plant leaves from different genera belonging to diverse families has been first isolated and pooled into three different groups as mentioned below, Control 2) simulated plant biomass controls (blended formulations) in which individual plant part having medicinal value has been mixed in three groups as control one and subjected to DNA isolation, and Control 3) genomic DNA (Isolated from plant leaves) pool from different species of the two genera (**Figure 1**). As mentioned above, three different groups were prepared for the first type of control with the plant species of a different genus. Group one comprised DNA of five species in equal proportion (5P), and further, in groups 2 and 3, DNA was added from ten (10P) and fifteen different plant species (15P) (**Figure 1**, **2A**). High-quality DNA of all the species have been isolated individually from leaf tissue and pooled together in equal proportion to make these groups. The group’s diversity has been increased by adding species from diverse genera that belong to diverse families to evaluate the resolution power and universality of the primers for the maximum number of species. For the second type of control, the same three groups of plants were used in the first controls (labeled as 5S, 10S, 15S). Still, simulated blended plant parts containing bioactive therapeutics were mixed in equal proportion (biomass admixture controls). These controls can be used to comprehend biases introduced during the DNA extraction and PCR dynamics under the influence of secondary metabolites on PCR amplification. For the third type of control, two groups were prepared. Group one comprises six plant species of the two genera, including *Asparagus* and *Terminalia* (**Figure 2B**). The second group includes seven plant species of the two genera, including *Piper* and *Phyllanthus* (**Figure 2B**). Similar to the first control, high-quality DNA was individually isolated from each species and pooled in equal amounts. These controls were utilized to obtain insight into the resolving strength of our newly designed *rbcL* and *ITS2* metabarcodes at lower taxa levels.

### 2.4 Metabarcoding

DNA from plant materials, blended formulations, and herbal products ‘was extracted in duplicate using DNeasy Plant Mini Kit (QIAGEN, Germany) following themanufacturer’s instructions. The library was prepared from each DNA sample using *rbcL* and *ITS2* fusion primers with optimized PCR conditions. The libraries were purified using AMPure XP beads (Beckman Coulter, CA, USA), and the quality of some of the libraries was checked using Agilent high-sensitivity DNA kit on Agilent 2100 Bioanalyzer. For each sample, libraries from two replicates were pooled. Further, all libraries were diluted to 100 pM and pooled in equimolar concentration. Emulsion PCR was carried out using Ion 520™ & Ion 530™ Kit-OT2 with 400 bp chemistry (Thermo Fisher Scientific, MA, USA). Sequencing was performed on the Ion S5 system using a 520/530 chip (Thermo Fisher Scientific, MA, USA). The data have been submitted to the National Centre for Biotechnology Information (NCBI) BioProject database under accession number PRJNA960808 (https://www.ncbi.nlm.nih.gov/bioproject/).

### 2.5 Metabarcoding data analysis

Optimization was done with three parameters for establishing the metabarcoding data analysis pipeline. 1) filtering criteria includes discarding reads with length <280 and >350 bp, <300 and >350 bp, <320 and >350 bp for *rbcL* and for *ITS2* discarding reads <280 and >300 bp; 2) OTU clustering with 97, 98 and 99% similarity in reads; 3) discarding OTU clusters having <5 and <10 reads. Obtained reads were filtered based on the quality score (Q >=25 for *rbcL* and Q >=20 for *ITS*) and read length using PRINSEQ (Schmieder and Edwards, 2011). Clustering filtered reads were performed using CD-HIT-EST (Huang et al., 2010). After that, the taxonomic assignment of OTU clusters having ≥5 and ≥10 reads was done using BLASTn (Altschul et al., 1990) (NCBI) with minimum E value 10E^-5^. For each sequence, ten hits were retrieved, and each hit was inspected and evaluated manually for the assigned plant genus and species. To analyze read abundance of each plant species, the number of reads was normalized by considering a total number of reads obtained after discarding clusters with <5 reads and <10 reads as 100%. The following formula was calculated for detection efficiency (%) for both metabarcodes. (Total number of detected targeted or labeled plant species/total number of plant species present in herbal formulation) × 100. Fidelity of detection (absolute) can be defined as the total number of samples or herbal formulations in which targeted plant species were detected per the total number of samples or herbal formulations in which targeted plant species were present (Seethapathy et al., 2019). The relative fidelity of detection (%) was calculated using the following formula. (Total number of samples or herbal formulations in which targeted plant species detected/total number of samples or herbal formulations in which targeted plant species are present) × 100. Fidelity of detection (absolute or relative) was calculated only where plant species that present in more than one group of the same type of control (n>1) and for market samples, sample size is more than one (n>1).

## 3 Results and discussion

Coghlan et al. (2012) first introduced a metabarcoding approach for detecting plant and animal raw materials used in 15 traditional Chinese medicines using a P-loop region of the plastid *trnL* gene and 16S mtDNA marker, respectively. Later, several studies reported application of metabarcoding for authentication and detection of plant materials in herbal medicines with single and multi-barcode approaches. For instance, Yao et al. (2022) and Cheng et al. (2014) employed a multi-barcode approach of *ITS2* and *trnL* in metabarcoding for detection of plant species in various traditional Chinese medicine (TCM). Urumarudappa et al. (2020) used *ITS2* and *rbcL* barcode in metabarcoding for detection of plant species in herbal medicines of Thailand. Using *ITS1* and *ITS2* barcodes in metabarcoding, Raclariu et al. (2017a; 2017b; 2018b) reported presence of unlabeled species by 89, 68, and 15% in single drugs of *Echinacea* species, *Hypericum perforatum*, and *Veronica officinalis*, respectively sold in the European market.

In 2014 and 2015, the total commercial market for herbal materials in India was estimated to be more than 512,000 tonnes, with a market value of USD 1 billion (Ved and Goraya, 2007). India has over 8,000 authorized medicinal product manufacturing units, and the market growth for herbal products is outstripping supply capacity for some plant species (Ved and Goraya, 2007). However, to date, detection of raw plant materials of Indian-marketed herbal medicine using metabarcoding approach is not well established. Ichim (2019) demonstrated 31% adulteration in 752 Indian-marketed herbal products with DNA barcoding and species-specific marker-based approach but not *via* metabarcoding. Earlier, we reported presence of unspecified plant species in four polyherbal formulations of the Indian market using *rbcL* minibarcode *via* metabarcoding approach (Pandit et al., 2021). Here, we have used a multi-barcode strategy to identify raw plant components in single drugs and polyherbal formulations of the Indian market using newly designed *rbcL* and *ITS2* metabarcodes.

### 3.1 PCR assays using newly designed *rbcL* and *ITS2* primers and fusion primers

Minimal criteria, such as universal amplifiabilities and minimum intraspecific but maximum interspecific divergence at the taxon level, must be followed in the search for the appropriate barcode region. Hence, degenerated *rbcL* and *ITS2* metabarcode primers were designed for high amplification efficiency, universality, and resolution power. In total, 45 medicinal plants from diverse families, genera, and species were taken to confirm and optimize the newly designed *rbcL* and *ITS2* primer sets for PCR amplification experimentally (**Supplementary Information Table S1**). The annealing temperature was optimized, and the results showed that the *rbcL* and *ITS2* primer sets performed optimum at 56°C (data not shown). Among newly designed *rbcL* and *ITS2* metabarcodes, *rbcL* is very robust and universal and gives a 100% amplification efficiency within selected 45 plants, but *ITS2* metabarcode gives 88.9% amplification efficiency and is not able to provide amplification in 5 plant species (11.1%) include *Ailanthus excelsa*, *Andrographis paniculata, A. vasica, O. tenuiflorum*, and *Ocimum canum* (**Supplementary Information Table S1**). However, due to the greater species-level discrimination power of *ITS2* in medicinal plants (Newmaster et al., 2013; Yao et al., 2022), *ITS2* metabarcode was taken together with *rbcL* metabarcode. The amplification effectiveness of “fusion primers” (tagged with Ion torrent adapter and barcodes) of *rbcL* and *ITS2* metabarcodes remained unchanged. However, the appearance of non-specific amplification in some barcodes suggests that 56°C annealing temperature is not optimal for the fusion primers. Therefore, further optimization of annealing temperature revealed that non-specific amplification was overcome by increasing the annealing temperature to 60°C (data not shown). At 60 °annealing temperature the amplification efficiency remained unaffected.

### 3.2 Establishing data analysis pipeline using mock controls

The first type of mock control, i.e., gDNA pooled controls of a different genus, was used to establish the metabarcoding data analysis pipeline. The first parameter is filtering criteria; for *ITS2*, the best filtering criteria was to remove reads with length < 300 bp, and for *rbcL*, discarding reads with length <300 and >350 bp (data not shown here). The second parameter is percentage similarity for the reads clustering (OTU picking), where in case of *rbcL* metabarcode, a greater number of plant species was detected when the reads were clustered at 99% identity. In the case of *ITS2* metabarcode, clustering the reads at 97% and 98% similarity were equally capable of resolving the plant species (**Supplementary Information Figure S1**). Therefore, for *ITS2* metabarcode, subsequently, for OTU clustering, i.e., 98% with a greater percentage was selected. The third parameter is the discarding OTU clusters having <5 or <10 reads, in which we observed that discarding OTU clusters having <5 reads was able to detect a greater number of plant species in the case of both metabarcodes (**Supplementary Information Figure S2**). Based on these findings, while selected read lengths were between 300 to 350 bp, 99% OTU clustering, and discarding of OTU clusters having <5 reads for the *rbcL* metabarcode and read lengths of >300 bp, 98% OTU clustering, and discarding of OTU clusters comprising <5 reads for the *ITS2* metabarcode for analyzing the metabarcoding data of other mock controls and commercial herbal formulations.

### 3.3 Metabarcoding of different types of mock controls

**Figure 3:**
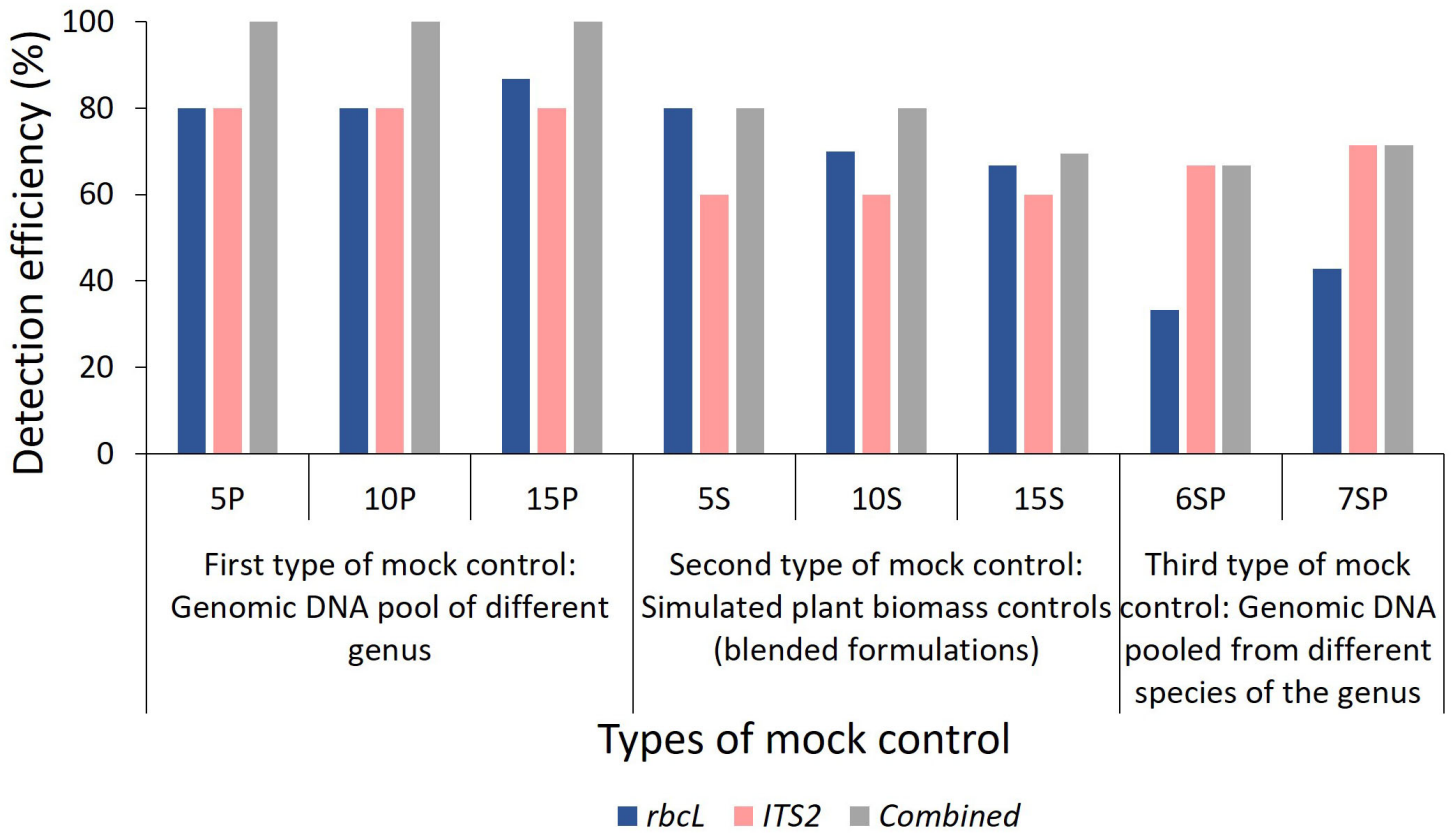
Detection efficiency obtained in mock controls by *rbcL*, *ITS2*, and combined approach. Detection efficiency (%) was calculated using a formula described in the Metabarcoding data analysis section of Materials and Methods. 5P: genomic DNA pool of five plant species, 10P: genomic DNA pool of ten plant species, 15P: genomic DNA pool of fifteen plant species, 5S: simulated blended plant pool of five plant species, 10S: simulated blended plant pool of ten plant species, 15S: simulated blended plant pool of fifteen plant species, 6SP: genomic DNA pool of six plant species from genera *Asparagus* and *Terminalia*, 7SP: genomic DNA pool of seven plant species from genera *Piper* and *Phyllanthus*. The detailed list of plant species used in each mock control is described in **Figure 2**.

Total reads obtained after filtering and percentage of reads analyzed (from filtered reads) after discarding OTU clusters having <5 reads for each mock control are shown in **Supplementary Information Table S2** for the first type of control, which is gDNA pooled controls of different genera comprising 5 (5P), 10 (10P), and 15 (15P) plant species total of 18657 and 452380 reads were obtained for *rbcL* and the *ITS2*, respectively (**Supplementary Information Table S2**). *Zingiber officinale* had the highest percentage of reads with *rbcL* metabarcode in 5P (45.9%) and 10P (30.9%), whereas *Senna tora* had the highest percentage (21.3%) in 15P (**Supplementary Information Figure S3A**). *Eclipta prostrata* had the highest percentage of reads with *ITS2* metabarcode in 5P (97.02%) and 10P (79.5%), whereas *Phyllanthus emblica* had the highest percentage (40.7%) in 15P (**Supplementary Information Figure S3A**). In 5P, out of five total four target plants were detected with *rbcL* metabarcode, and three were detected with *ITS2*. In 10P, out of ten, nine targeted plants were detected with *rbcL*, and seven targeted plants were detected with *ITS2*. In 15P, out of fifteen, thirteen targeted plants were detected with *rbcL*, and twelve targeted plants were detected with *ITS2* (**Figure 2**; **Supplementary Information Figure S3A**). On the whole, for the gDNA pooled controls of a different genus, detection efficiency of *rbcL* was observed at 80% for 5P and 10P, 86.7% for 15P. While detection efficiency of *ITS2* was observed at 80%, and combined detection efficiency of both metabarcodes was observed at 100% for all three gDNA pooled mock controls (**Figures 2A**, **3**). Five plant species in all three gDNA pooled mock controls had 80% average fidelity, and the other five were present in two groups, i.e., 10P and 15P, had 90% average fidelity with *rbcL* metabarcode (**Table 2**). *ITS2* metabarcode exhibited 66.7% average fidelity for five plant species present in all three gDNA pooled mock controls and 90% average fidelity for other five plant species present in 10P and 15P. Combined average fidelity with both barcodes was 100% for gDNA pooled control (**Table 2**).

**Table 2:** Detection fidelity in mock controls^≠^.

Sr. no.	Plant Species	gDNA pooled mock controls				Simulated plant biomass controls (Blended formulations)			
			Relative fidelity/species (%)				Relative Fidelity/species (%)		
		Species present in no. of mock controls^#^	*rbcL*	*ITS2*	Combined	Species present in no. of mock controls^®^	*rbcL*	*ITS2*	Combined
1	*Andrographis paniculata*	3	100	0	100	3	100	0	100
2	*Azadirachcta indica*		100	33.3	100		100	66.7	100
3	*Eclipta alba*		100	100	100		100	100	100
4	*Piper nigrum*		0	100	100		100	100	100
5	*Zingiber officinale*		100	100	100		0	0	0
**Average Fidelity (%)**			**80**	**66.7**	**100**		**80**	**53.3**	**80**
6	*Aegale marmelous*	2	100	50	100	2	100	50	100
7	*Centella asiatica*		100	100	100		100	100	100
8	*Terminalia arjuna*		50	100	100		0	0	0
9	*Terminalia bellerica*		100	100	100		50	100	100
10	*Vitex negundo*		100	100	100		100	100	100
**Average Fidelity (%)**			**90**	**90**	**100**		**70**	**70**	**80**

^#^3: Species present in all three groups of gDNA pooled mock control (n=3); 2: Species present in two groups of gDNA pooled mock control i.e., 5P and 10P (n=2). ^®^3: Species present in all three groups of simulated plant biomass mock control (n=3); 2: Species present in two groups of simulated plant biomass mock control i.e., 5S and 10S (n=2). ^#®^Fidelity was not calculated for five plant species that present only in 15P and15S (n=1). **^≠^** Fidelity for the third type of mock control (i.e., gDNA pooled from different species of the genus) was not calculated as each plant species present only in one group (n=1).

For the simulated plant biomass controls (i.e., blended formulations or second type of mock controls), 42140 and 334017 reads were obtained after filtering for *rbcL* and the *ITS2*, respectively (**Supplementary Information Table S2**). The highest percentage of reads was observed for *Azadirachta indica* in 5S (62.5%) and 10S (34.7%), whereas, in 15S *Justicia adhatoda* (24.8%) showed highest percentage of reads using *rbcL* metabarcode (**Supplementary Information Figure S3B**). While in the case of *ITS2*, *Eclipta prostrata* had the highest percentage of reads in all simulated plant biomass controls (**Supplementary Information Figure S3B**). In 5S, 10S, and 15S total of 3, 7, and 10 targeted plants were detected by *rbcL*, and a total of 3, 6, and 8 targeted plants were detected by *ITS2*, respectively (**Figure 2A**; **Supplementary Information Figure S3B**). Detection efficiency for simulated plant biomass controls was observed 80% for 5S, 70% for 10S, and 66.7% for 15S with *rbcL* metabarcode. While detection efficiency of *ITS2* was observed at 60% for all three groups of simulated plant biomass control, and combined detection efficiency of both metabarcodes was observed 80% for 5S and 10S and 69.4% for 15S (**Figure 3**). Five plant species that were present in all three groups of simulated plant biomass control had 80% average fidelity, and other five plant species that were present in 10S and 15S had 70% average fidelity with *rbcL* metabarcode (**Table 2**). *ITS2* metabarcode exhibited 53.3% average fidelity for five plant species in all three groups and 70% average fidelity for five other plants present in two groups, i.e., 10S and 15S. Combined average fidelity with both barcodes was observed at 80% for simulated plant biomass controls (**Table 2**).

For the third type of control, gDNA pooled from different species of two genera, 10265 and 164358 reads were obtained for *rbcL* and the *ITS2*, respectively (**Supplementary Information Table S2**). In 6SP, out of six plant species total of three plant species include *Asparagus racemosus* (81.1%), *Terminalia bellirica* (11.8%), and *Terminalia chebula* (6.8%), were detected by *rbcL* while *ITS2* metabarcode was able to resolve five plant species except for *Asparagus dumosus* (**Figure 2B**; **Supplementary Information Figure S3C**). In 7SP, out of seven plant species, two plant species include *Piper nigrum* (24.7%) *and Phyllanthus emblica* (72.3%) were detected by **rbcL*. In comparison, *ITS2** metabarcode was able to resolve four plant species, including *Piper nigrum* (0.8%)*, Piper longum* (21%)*, Phyllanthus emblica* (62.5%)*, Phyllanthus amaras* (13.5%) (**Figure 2B**; **Supplementary Information Figure S3C**). *rbcL* showed 33.3%, and *ITS2* showed 66.7% detection efficiency in species-level control with a combined detection efficiency of 66.7% (**Figures 2B**, 2). These two species-level controls indicate a greater resolution spectrum of *ITS2* metabarcode than *rbcL* metabarcode. This finding corroborates with earlier reports in which authors demonstrated that *ITS2* metabarcode has greater species-level discrimination power than *rbcL* while *rbcL* has greater universality (Newmaster et al., 2013; Yao et al., 2022).

Here, in the first and third types of mock controls, DNA was pooled in equal proportions, and for the second type of simulated plant biomass controls, the equivalent weight of each plant species part with therapeutic importance was mixed. The primary aim of all three mock controls was to evaluate the read abundance, detection efficiency, and fidelity differences introduced under the influence of secondary metabolites, primer fit compatibilities, and PCR dynamics. The impact was observed with percentage read variation of the same plant in a different control (Arulandhu et al., 2017). *Terminalia arjuna*, *T. chebula*, and *Phyllanthus emblica* were not detected in simulated plant biomass control might be due to variability in quality and quantity of DNA extracted from each plant species from the mixtures as different parts of plants, i.e., rhizome, fruits, leaves, and bark have been added into plant biomass controls (Ivanova et al., 2016). In addition to that, *ITS2* metabarcode is unable to resolve *A. paniculata* and *J. adhatoda* in all mock controls because the newly designed *ITS2* metabarcode is impotent in amplifying target sequence from these two plant species (**Figure 1**; **Supplementary Information Table S1**). Despite the mentioned limitations, the combined *rbcL* and *ITS2* metabarcoding approach could resolve plant species with high fidelity (**Table 2**) and can be implemented to detect plant species in herbal products.

### 3.4 Metabarcoding of single drugs

Tulsi (*O. tenuiflorum*) (The Ayurvedic Pharmacopoeia of India, Vol. II, 1999), Gokhru (*Tribulus terrestris*) (The Ayurvedic Pharmacopoeia of India, Vol. I,1990) Shatavari (*Asparagus racemosus*) (The Ayurvedic Pharmacopoeia of India, Vol. III, 2001), Vasa (*Justicia adhatoda*), Ashwagandha (*Withania somnifera*), Bhringraj (*Eclipta alba*), and Arjuna (*Terminalia arjuna*) were among the 17 single drugs that were collected. Total reads, reads obtained after filtering, and percentage of analyzed reads (from filtered reads) after discarding OTU clusters having <5 reads for each single drug are shown in **Supplementary Information Table S3**. For all single drugs, 135505 total raw reads for *rbcL* and 1390098 total raw reads for *ITS2* metabarcode were obtained (**Supplementary Information Table S3**). On an average, 12.6% (0-99.8%) reads have been obtained for non-targeted plant species with *rbcL* metabarcode and 46.5% (0-100%) reads have been obtained for non-targeted plant species with *ITS2* metabarcode in single drugs (**Supplementary Information Table S4**). Cross-contamination with allied species, harvesting process, pollen contamination, misidentification due to cryptic taxonomy, polynomial vernacular identification, manufacturing and packing procedure may contribute to the presence of non-targeted plant species (Seethapathy et al., 2019).

**Figure 4:**
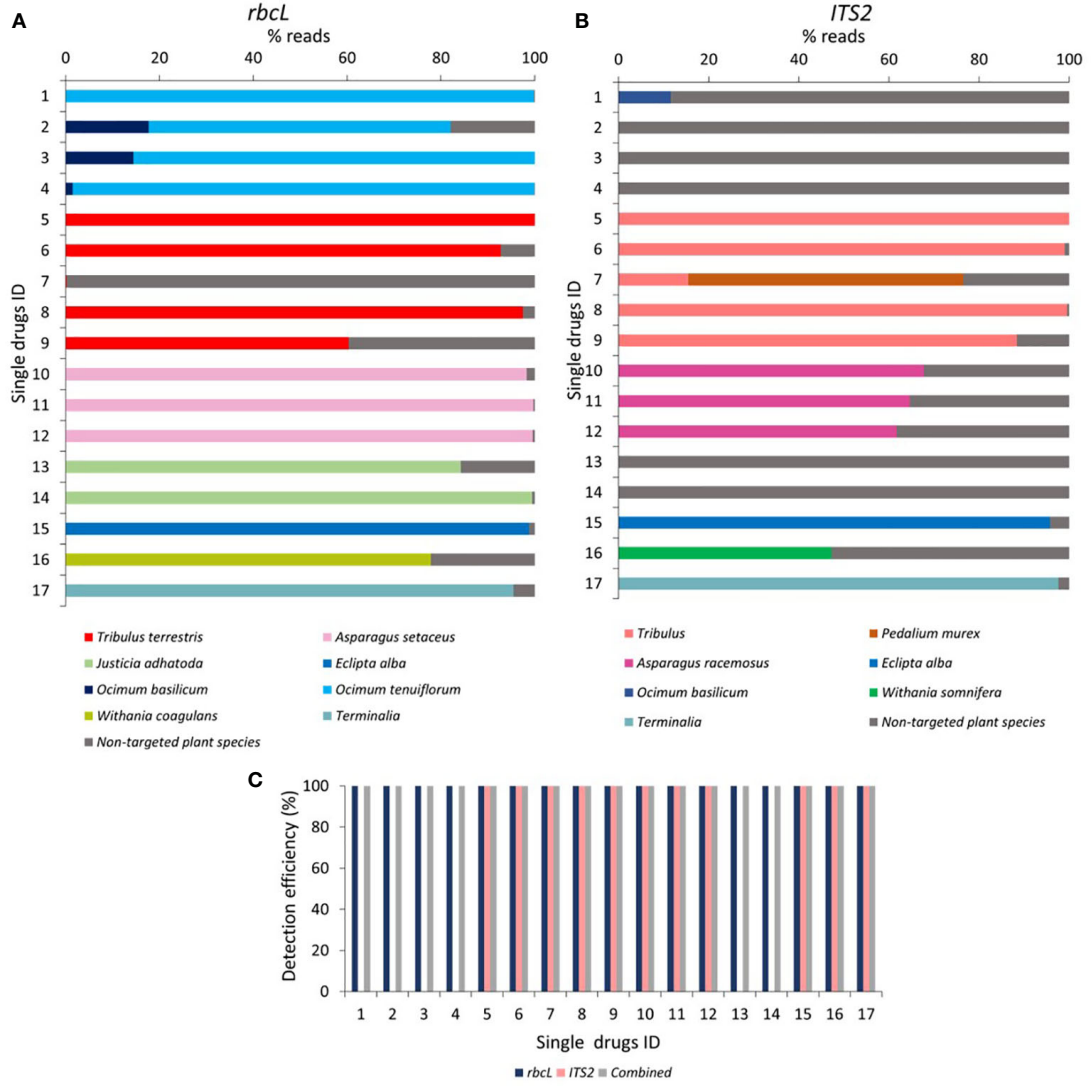
Relative abundance of the plant species and detection efficiency in single drugs through *rbcL* and *ITS2* metabarcoding. **(A)** Relative abundance (% reads) of the plant species detected in single drugs through *rbcL* metabarcoding. Relative abundance (% reads) of non-targeted plant species is reported in **Supplementary Information Table S4**. **(B)** Relative abundance (% reads) of the plant species detected in single drugs through *ITS2* metabarcoding. The relative abundance of non-targeted plant species is reported in **Supplementary Information Table S4**. **(C)** Detection efficiency obtained in single drugs by *rbcL*, *ITS2*, and combined metabarcoding approach. Single drugs ID 1 to 4 for Tulsi (*Ocimum tenuiflorum*) powder, 5 to 9 for Gokhru (*Tribulus terrestris*) powder, 10 to 12 for Shatavari (*Asparagus racemosus*) powder, 13 and 14 for Vasa (*Justicia adhatoda*) powder, 15 for Bhringraj (*Eclipta alba*) powder, 16 for Ashwagandha (*Withania somnifera*) powder, and 17 for Arjuna (*Terminalia arjuna*) powder.

In tulsi powder (labeled as *O.tenuiflorum*), *rbcL* detected *O.tenuiflorum* ranging between 99.8% to 64.4%. Along with *O. tenuiflorum*, substituted *Ocimum basilicum* (Travadi et al., 2022b) was also observed in three herbal products with 17.7, 14.4, and 1.4% reads with *rbcL* metabarcode, respectively (**Figure 4A**). *O. tenuiflorum* could not be detected by *ITS2* metabarcode in any samples. However, *O. basilicum* was found in one sample with 11.6% reads (**Figure 4B**). That could be due to inability of new *ITS2* metabarcode to amplify *O. tenuiflorum* (**Supplementary Information Table S1**), which is proof of PCR biases toward the unintentional and low level of contamination present in samples and leads to the high number of reads for non-targeted plant species.

In Gokhru powder, *T.terrestris* (Chota Gokhru) was detected up to species level by *rbcL* with 100% reads in sample 5, 92.7% in sample 6, 0.2% in sample 7, 97.4% in sample 8, and 60.3% in sample 9 (**Figure 4A**). The *ITS2* metabarcode resolved *T. terrestris* only at the genus level with 100% in sample 5, 99% in sample 6, 15.5% in sample 7, 99.6% in sample 8, and 88.4% in sample 9. *ITS2* revealed the presence of 61% of *Pedalium murex* (Bada Gokhru) in sample 7 (**Figure 4B**), which is a commonly substituted species and labeled as Gokhru in Indian marketed herbal products (Choudhary et al., 2021).

In all three Shatavari powders, *rbcL* metabarcode detected *Asparagus setaceus* instead of *A. racemosus* with 98.3% reads in sample 10, 99.7% in sample 11, and 99.6% reads in sample 12. By *ITS2*, 67.7% to 61.7% reads for *A. racemosus* were obtained in all three Shatavari powders. In vasa powder, *J.adhatoda* was detected by *rbcL* with 84.2% reads in sample 13 and 99.4% in sample 14. In comparison, *ITS2* could not resolve *J.adhatoda* because of the amplification inability of our *ITS2* metabarcode (**Figure 4**, **Supplementary Information Table S1**). This result was consistent with the mock control 15P and 15S. In Ashwagandha powder (sample 16), *Withania coagulans* (77.9% reads) instead of *W. somnifera* was detected by *rbcL*. While with *ITS2*, *W. somnifera* was detected with 47.2% reads.

In Bhringraj powder, both *rbcL* and *ITS2* metabarcode detected *E.alba* with 98.8% and 95.7% reads, respectively. In Arjuna powder, *rbcL* metabarcode identified *Terminalia arjuna* only up to genus level with 95.5% reads, while *ITS2* metabarcode was identified *T. arjuna* at species level with 97.6% reads (**Figure 4**).

For Tulsi, Gokhru, Shatavari, and Vasa powder, 100% fidelity was obtained with *rbcL* metabarcode, while *ITS2* metabarcode exhibited 0% fidelity for Tulsi and Vasa powder and 100% fidelity for Gokhru and Shatavari powder (**Table 3A**). Suggesting that, for Tulsi and Vasa powder authentication, our *rbcL* metabarcode works efficiently but not *ITS2*. While *rbcL* metabarcode was not suitable for identifying P. murex in Gokhru powder, *rbcL* sequence for *Pedalium murex* was unavailable in NCBI (database was accessed on December 15, 2022). In addition, *T. arjuna* was resolved only up to genus level by *rbcL*, and *T. terrestris* was resolved up to only at the genus level by *ITS2* metabarcode. This observation is in concordance with mock controls and could be due to low interspecific variability of barcode sequence covered by our metabarcodes for resolving species to be identified. Although, combined metabarcoding approach provides 100% detection efficiency with 100% fidelity for single drugs by overcoming the limitations of individual barcodes due to PCR biases, low interspecific variability, or the absence of the corresponding sequences in the database. Seethapathy et al. (2019) demonstrated 67% fidelity for targeted plant species present in single drugs of the European market using *ITS1* and *ITS2* barcodes. We obtained 100% fidelity for targeted plant species within single drugs. The overall results of the single drugs revealed that a multi-barcode metabarcoding approach could be used to assess the prevalence of widespread adulterated and substituted plant material in single drugs and implement more stringent supply chain precautionary measures at primary level.

Table 3Detection fidelity of single drugs and polyherbal formulations.

**Table 3a:** Detection fidelity of single drugs.

Name of Herbal product	Composition of formulations	No. of products	Absolute fidelity^#^ /single drug			Relative fidelity (%)/single drug		
			*rbcL*	*ITS2*	Combined	*rbcL*	*ITS2*	Combined
Tulsi	*Ocimum tenuiflorum*	4	4	0	4	100	0	100
Gokhru	*Tribulus terrestris*	5	5	5	5	100	100	100
Shatavari	*Asparagus racemosus*	3	3	3	3	100	100	100
Vasa	*Justicia adhatoda*	2	2	0	2	100	0	100
**Cumulative fidelity of single drugs (%)**						**100**	**50**	**100**

^#^Fidelity was not calculated for Bhurgraj, Ashawgandha and Arjuna where n=1.

**Table 3b:** Detection fidelity of polyherbal formulations.

Name of Herbal product	Composition of formulations	No. of products	Absolute fidelity of detection/species/polyherbal formulation^≠^			Relative fidelity of detection/ species/polyherbal formulation			Average relative fidelity (%)/ polyherbal formulations		
			*rbcL*	*ITS2*	Combined	*rbcL*	*ITS2*	Combined	*rbcL*	*ITS2*	Combined
Trikatu	*Zinger officinale*	3	3	3	3	100	100	100	88.9	100	100
	*Piper nigrum*		3	3	3	100	100	100			
	*Piper longum*		2	3	3	66.7	100	100			
Sitopaladi	*Piper logum*	3	3	3	3	100	100	100	77.8	66.7	77.8
	*Eletaria cardamom*		3	3	3	100	100	100			
	*Cinnamomum cassia*		1	0	1	33.3	0	33.3			
Rasayana	*Tribulus terrestris*	4	4	4	4	100	100	100	75	33.3	75
	*Tinospora sinensis*		4	0	4	100	0	100			
	*Phyllanthus emblica*		1	0	1	25	0	25			
Hingwashtak	*Zinger officinale*	4	4	1	4	100	25	100	57.1	39.3	63.3
	*Piper nigrum*		2	0	2	50	0	50			
	*Piper longum*		1	2	2	25	50	50			
	*Apium graveolens* (substituted with *Trachyspermum ammi*)		4	3	4	100	75	100			
	*Cyminum cyminum*		4	4	4	100	100	100			
	*Carum carvi* [substituted with *Bunium persicum (Elwendia persica)]*		0	1	1	0	25	25			
	*Ferula foetida*		1	0	1	25	0	25			

^≠^Fidelity was not calculated for Talisadi/Talisadya as n=1.

### 3.5 Metabarcoding of polyherbal formulations

Trikatu, Sitopaladi, Rasayana, Hingwashtak, and Talisadi (Talisadya) (Joshi et al., 2017) were among the 15 polyherbal formulations collected. Total reads obtained after filtering and percentage of analyzed read (from filtered reads) after discarding OTU clusters having <5 reads for each polyherbal formulation are shown in **Supplementary Information Table S5**. A total of 53087 and 1429238 reads were obtained by *rbcL* and *ITS2* metabarcode for polyherbal formulation, respectively (**Supplementary Information Table S5**). On average, 1.4% (0-3.9%) reads have been obtained for non-targeted plant species with *rbcL* metabarcode, and 16.5% (0.1-87.4%) reads were obtained for non-targeted plant species with *ITS2* metabarcode in polyherbal formulations (**Supplementary Information Table S6**).

**Figure 5:**
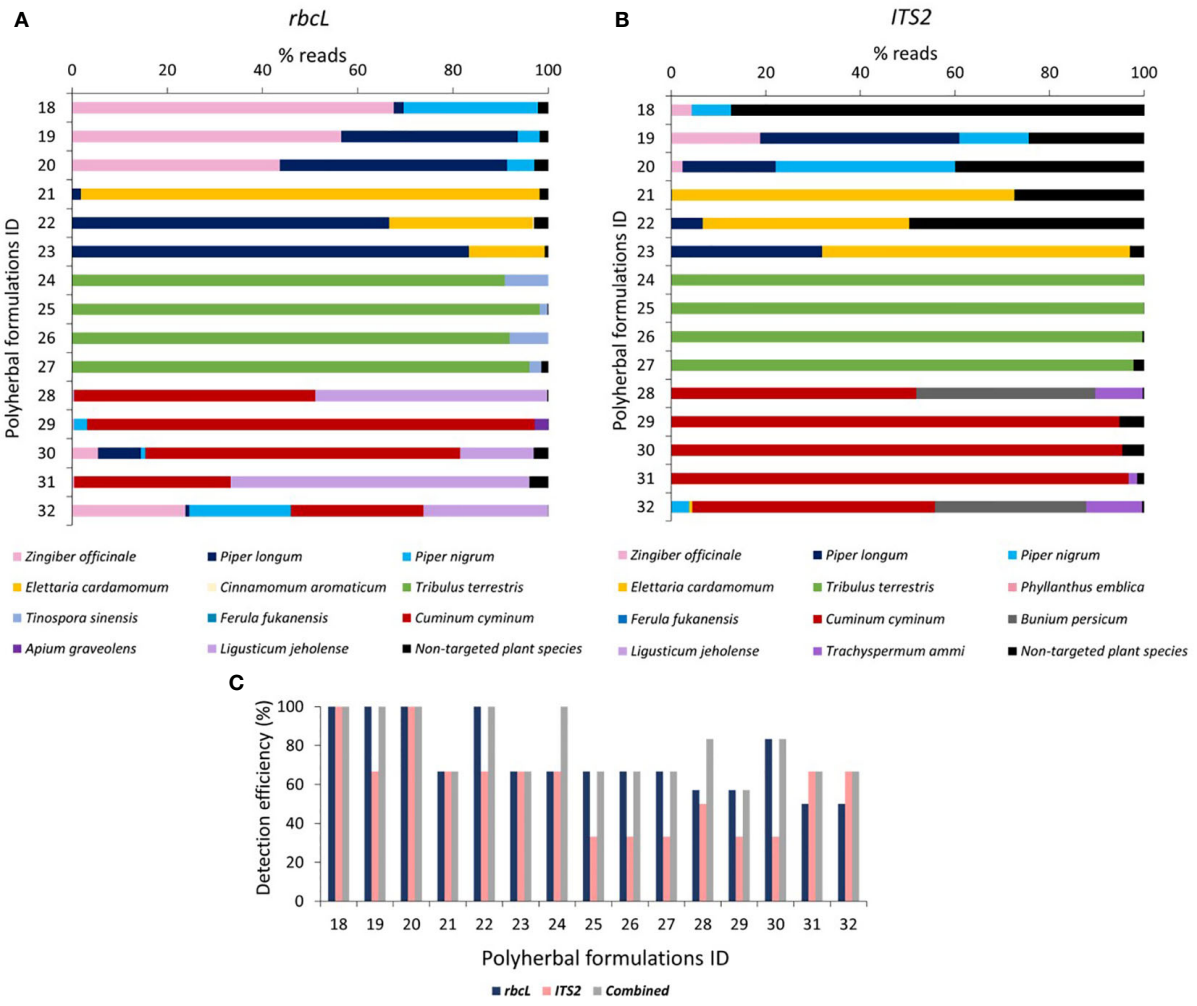
Relative abundance of the plant species and detection efficiency in polyherbal formulations through *rbcL* and *ITS2* metabarcoding. **(A)** Relative abundance (% reads) of the plant species detected in polyherbal formulations through *rbcL* metabarcoding. Relative abundance (% reads) of non-targeted plant species is reported in **Supplementary Information Table S6**. **(B)** Relative abundance (% reads) of the plant species detected in polyherbal formulations through *ITS2* metabarcoding. Relative abundance (% reads) of non-targeted plant species is reported in **Supplementary Information Table S6**. **(C)** Detection efficiency obtained in polyherbal formulation by *rbcL*, *ITS2*, and combined metabarcoding approach. Polyherbal formulations ID 18 to 20 for Trikatu powder, 21 to 23 for Sitopaladi powder, 24 to 27 for Rasayana powder, 28 to 31 for Hingwashtak powder, and 32 for Talisadi powder.

In Trikatu, 28.2%, 4.6%, and 57% read for *P. nigrum* with *rbcL*, and 8.3%, 14.7 and 37.9% reads with *ITS2* was observed in sample 18, 19, and 20, respectively. *P.longum* comprised 2%, 37.1%, and 47.8% reads with *rbcL* and 0%, 42.1%, and 19.7% with *ITS2*; *Z. officinale* possess 67.6%, 56.5%, and 43.6% with *rbcL* and 4.3%, 18.8% and 2.4% with *ITS2* in sample 18, 19, and 20 respectively (**Figures 5A, B**). All three targeted plants were detected (i.e., 100% detection efficiency) in all three Trikatu samples (i.e., 100% fidelity) using a combined approach (**Figure 5C**, **Table 3B**). Nevertheless, *ITS2* showed the higher percentage of non-targeted reads (87.4% in sample 18, 24.4% in sample 19, and 40% in sample 20) might be due to technical bias that can be introduced during DNA extraction and PCR towards the unintentional cross-contamination happens during the supply chain (**Figure 5B**).

Sitopaladi powder is primarily composed of five constituents; with the exclusion of sacrarium (sugar) and vanshlochan (the female bamboo exudate) and from the total number of designated species, the aim was to detect *C. cassia*, *P. longum*, and *Elettaria cardamom*. *C. cassia* was detected by *rbcL* metabarcode with 0.2% reads only in sample 22. P*. longum* exhibited 1.8, 66.6, and 83.3% of reads with *rbcL* and 0.1, 6.6, and 31.9% of reads with *ITS2* in samples 21, 22, and 23, respectively. *E. cardamomum* showed 96.3%, 30.2%, and 15.9% reads with *rbcL* and 72.4%, 43.7%, and 65.1% with *ITS2* in samples 21, 22, and 23, respectively (**Figures 5A, B**). Overall, the combined approach showed 66.7% detection efficiency in sample 21, 100% in sample 22, and 66.7% in sample 23, and average of 77.8% fidelity (**Figure 5C**, **Table 3B**).

In Rasayana, *rbcL* metabarcode exhibited 91% to 98% reads for *T. teresteris*, while *ITS2* metabarcode exhibited 98 to 100% reads in all samples (samples 24 to 27). *In all samples, T. cordifolia was resolved by *rbcL** with 1.7% to 9.1% reads, while *ITS2* metabarcode could not (**Figures 5A, B**). *P. emblica* was not detected by both metbarcode except in sample 24 (**ITS2* obtained 0.01% reads were)*, possibly due to DNA extraction biases as DNA extraction from amla fruits is difficult due to high acidic nature and high tannin content (Warude et al., 2003). The combined metabarcoding approach showed 100% detection efficiency in sample 24 and 66.7% in remaining other samples with average of 75% fidelity (**Figure 5C**, **Table 3B**).

Hingwashtak powder (samples 28 to 31) comprised seven ingredients, including *Zinger officinale, P. nigrum, P. longum, C. cyminum, C. carvi* [*C. cyminum, and C. carvi* commonly substituted with *Bunium persicum* (Syn. *Elwendia persica*) (Johri, 2011; Singh et al., 2017; Bansal et al., 2018)]*, Apium leptophyllum* (majority of commercial products comprised/labeled *Apium graveolens* instead of *A. leptophyllum*; further these plant species commonly substituted with *T. ammi* (Pushpendra et al., 2016)) and *Ferula foetida.* From these seven ingredients, *rbcL* metabarcode was able to resolve *Z. officinale* in all samples with 0.37 to 5.5% reads, *P. nigrum* in sample 29 (2.7% reads) and 30 (1.0% reads)*, P. longum* in sample 30 (8.9% reads), *C. cyminum* in all samples with 32.7% to 94.1% reads, *A. graveolens* in sample 29 (2.9% reads). *C. carvi* commonly substituted with *B. persicum* (*Elwendia persica*), neither *C. carvi* nor *B. persicum* was detected by *rbcL* in all Hingwashtak samples (**Figure 5A**). *ITS2* metabarcode exhibited a high prevalence of *C. cyminum* in all samples with 51.8% to 95.4% reads, *B. persicum* in sample 28 with 37.9% reads, *T. ammi* (substitution of *A. leptophyllum* or *A. graveolens*) in sample 28 with 10% reads (**Figure 5B**). In addition, *ITS2* metabarcode also detected Trachyspermum ammi in samples 29 and 31*. rbcL* metabarcode showed reads for *Ligusticum jeholense* (Chinese medicinal herb from the Apiaceae family) instead of *Trachyspermum ammi* in sample 28, 30, 31 and *A. graveolens* in sample 29. This could be due to our *rbcL* metabarcode unable to resolve *T. ammi* and detect *L. jeholense* falling under the same family. Overall, the combined metabarcoding approach showed average 72.6% detection efficiency with 63.3% fidelity for Hingwashtak powders (**Figure 5C** and **Table 3B**).

Talisadi/Talisadya powder (sample 32) comprises eight constituents including *Abies webbiana, P. longum, P. nigrum, Z. officinale, E. cardamomum, Cinnamomum Zeylanicum*, Vanshlochan (the female bamboo exudate), and sugar (sugar and Vanshlochan are excluded for metabarcoding analysis). From these six ingredients, only three plant species which include *P. longum* (0.8% reads)*, P. nigrum* (21.3% reads), and *Z. officinale* (23.8% reads) were detected by *rbcL* metabarcode. Three plant species which include *P. nigrum* (3.79% reads)*, Z. officinale* (0.03% reads), and *E. cardamomum* (0.6% reads) were detected by *ITS2* metabarcode. *A. webbiana* and *C. Zeylanicum* were not detected by either of the barcodes. The combined metabarcode approach detected four plant species (66.7% detection efficiency) out of six (**Figure 5C**). In Talisadi/Talisadya powder, *C. cyminum* (27.9% reads with *rbcL* and 51.3% reads with *ITS2*), *B. persicum* (32.0% reads with *ITS2*), *L. jeholense* (26.2% reads with *rbcL*) and *T. ammi* (11.8% reads with *ITS2*) was detected might be due to unintentional cross-contamination happen during sample processing as a collection of Hingwashtak powder sample 28 and Talisadi powder sample 32 were done from the same company. In addition, a high percentage of reads were covered by *C. cyminum*, *T. ammi*, *L. jeholen*s, *B. persicum* (all plant species belonging to Apiaceae family), then *Z. officinale*, *P. longum*, and *P. nigrum*. That could be because of technical bias introduced during DNA extraction and PCR.

### 3.6 Fidelity of targeted plant species

Up to this point, the fidelity of plant species per number of mock controls or herbal formulations has been calculated and discussed. Here, we have estimated the fidelity of the targeted plant species included within different mock controls and polyherbal formulations to get a better perspective of species discrimination capabilities and reliabilities of single and multi-barcode approaches. Both the *rbcL* and *ITS2* metabarcodes resolved 19 (46.7%) of the 39 listed plant species at the species level. However, plants detected at the species level were different, and a multi-barcode approach provided species-level resolution for 27 (69.23%) species, leading to a 20.5% increment in the whole (**Table 4**). This observation confirms robustness for our newly designed metabarcodes in detecting plant species at lower taxonomic levels. In addition, 100% fidelity was observed for *T. bellirica, A. paniculata, A. indica, E. alba*, and *C. asiatica* within gDNA controls, biomass controls, and cumulative analysis by the combined approach of *rbcL* and *ITS2*. However, the combined approach of *rbcL* and *ITS2* exhibited 100% fidelity for *Z. officinale*, *V. negundo*, *P. nigrum*, *A. marmelous*, *T. arjuna*, and *P. embilica* only within gDNA controls and having lower fidelity in biomass controls and cumulative analysis (**Table 4**). That could be due to biases in the DNA isolation process; yielding equal proportional DNA from the poly formulation is impossible due to genome size differences and differences in plant parts and amounts of secondary metabolites.

**Table 4:** Fidelity of targeted plant species present within mock controls as well herbal formulations.

Plant species	Family	Plant part used in simulated biomass mock control or formulations	Resolution at taxa level		A number of gDNA mock controls in which plant species present	Relative fidelity of each plant species presents within first and third type of mock controls (gDNA controls)			A total number of biomass controls, single drugs and polyherbal formulations in which plant species present	Relative fidelity of each plant species presents within second type of mock control (simulated biomass control), single drugs and polyherbal formulations (biomass controls + herbal formulations)			A number of mock controls and herbal products in which plant species present	Relative fidelity of each plant species presents in different types of mock controls and herbal products (cumulative analysis)		
			*rbcL*	*ITS2*		*rbcL*	*ITS2*	Combined		*rbcL*	*ITS2*	Combined		*rbcL*	*ITS2*	combined
*Andrographis paniculata*	Acanthaceae	Whole plant	Species	ND	3	100	0	100	3	100.0	0.0	100.0	6	100.0	0.0	100.0
*Azadirachcta indica*	Meliaceae	Leaves	Species	ND	3	100	33.3	100	3	100.0	66.7	100.0	6	100.0	50.0	100.0
*Eclipta alba*	Asteraceae	Whole plant	Species	Species	3	100	100	100	4	100.0	100.0	100.0	7	100.0	100.0	100.0
*Piper nigrum*	Piperaceae	Seed	Species	Species	4	100	100	100	11	81.8	90.9	90.9	15	86.7	93.3	93.3
*Zingiber officinale*	Zingiberaceae	Rhizome	Family	Family	3	100	100	100	11	54.5	63.6	63.6	14	64.3	71.4	71.4
*Aegale marmelous*	Rutaceae	Fruits	Family	ND	2	100	50	100	2	50.0	0.0	50.0	4	75.0	25.0	75.0
*Centella asiatica*	Apiaceae	Whole plant	Species	Species	2	100	100	100	2	100.0	100.0	100.0	4	100.0	100.0	100.0
*Terminalia arjuna*	Combretaceae	Bark	Genus	Species	3	0	100	100	3	33.3	33.3	33.3	6	16.7	66.7	66.7
*Terminalia bellerica*	Combretaceae	Fruits	Species	Species	3	100	100	100	2	0.0	100.0	100.0	5	60.0	100.0	100.0
*Vitex negundo*	Lamiaceae	Leaves	Species	species	2	100	100	100	2	50.0	50.0	50.0	4	75.0	75.0	75.0
*Bacopa monneri*	Plantaginaceae	Whole plant	Species	Species	1	NA	NA	NA	1	NA	NA	NA	2	50.0	100.0	100.0
*Cassia tora*	Fabaceae	Leaves	Species	Species	1	NA	NA	NA	1	NA	NA	NA	2	50.0	100.0	100.0
*Phyllanthus embilica*	Phyllanthaceae	Fruits	Species	Species	2	100	100	100	5	0.0	20.0	20.0	7	28.6	42.9	42.9
*Terminalia chebula*	Combretaceae	Fruits	Genus	Species	2	100	100	100	1	NA	NA	NA	3	66.7	66.7	100.0
*Justicia adhatoda*	Acanthaceae	Leaves	Species	ND	1	NA	NA	NA	3	100.0	0.0	100.0	4	100.0	0.0	100.0
*Piper longum*	Piperaceae	Fruits	Species	Species	1	NA	NA	NA	11	63.6	72.7	72.7	12	58.3	75.0	75.0
*Asparagua racemosus*	Asparagaceae	Root	Genus	Species	1	NA	NA	NA	3	100.0	100.0	100.0	4	100.0	100.0	100.0
*Tribulus terrestris/* *Pedalium murex*	Zygophyllaceae/Pedaliaceae	Fruits	Species/ ND	Genus/ Species					9	100.0	100.0	100.0	9	100.0	100.0	100.0
*Ocimum tenuiflorum/* *Ocimum basilicum*	Lamiaceae	Leaves	Species/ Species	ND/ND					4	100.0	0.0	100.0	4	100.0	0.0	100.0
*Elettaria cardamomum*	Zingiberaceae	Seed	Species	Species					4	75.0	100.0	100.0	4	75.0	100.0	100.0
*Carum carvi/* *Elwendia persica*	Apiaceae	Seed	ND/ND	ND/ Genus					4	0.0	25.0	25.0	4	0.0	25.0	25.0
*Cinnamomum cassia*	Lauraceae	Bark	Genus	ND					4	25.0	0.0	25.0	4	25.0	0.0	25.0
*Cyminum cyminum*	Apiaceae	Seed	Species	Species					4	100.0	100.0	100.0	4	100.0	100.0	100.0
*Ferula foetida*	Apiaceae	Gum resin	ND	Genus					4	0.0	25.0	25.0	4	0.0	25.0	25.0
*Tinospora sinensis*	Menispermaceae	Root	Species	ND					4	100.0	0.0	100.0	4	100.0	0.0	100.0
*Apium leptophyllum* */Trachyspermum ammi/Apium graveolens*	Apiaceae	Seed	ND/ND/Species	ND/ Species/ND					4	100.0	75.0	100.0	4	100.0	75.0	100.0
*Withania somnifera*	Solanaceae	Root	Genus	Species					1	NA	NA	NA	1	NA	NA	NA
*Abies webbiana*	Pinaceae	Leaves	ND	ND					1	NA	NA	NA	1	NA	NA	NA
*Asparagua dumosus*	Asparagaceae	Leaves	ND	ND	1	NA	NA	NA					1	NA	NA	NA
*Asparagus adscendens*	Asparagaceae	Leaves	ND	Species	1	NA	NA	NA					1	NA	NA	NA
*Phyllanthus amaras*	Phyllanthaceae	Leaves	ND	Species	1	NA	NA	NA					1	NA	NA	NA
*Phyllanthus madresapatensis*	Phyllanthaceae	Leaves	ND	ND	1	NA	NA	NA					1	NA	NA	NA
*Piper betle*	Piperaceae	Leaves	ND	ND	1	NA	NA	NA					1	NA	NA	NA
*Pipper arabarum*	Piperaceae	Leaves	ND	ND	1	NA	NA	NA					1	NA	NA	NA

ND: Not detected, NA: Species not applicable for fidelity calculation as present only either in one mock control or either in one herbal formulations (n=1). Plant species represent in red colour are substituted plant species.

Furthermore, the extracted DNA is degraded because herbal products are intensively processed. The PCR conditions and reactions will also have a significant impact on the primer fit and PCR bias of the mixture. That was demonstrated by comparing the combined fidelity of gDNA controls, biomass controls, and cumulative analysis (**Table 4**). On average, 83.6% fidelity was observed for targeted plant species in the cumulative analysis. This result confirmed the high reliability of our multi-barcode sequencing approach.

## 4 Conclusion

On the whole, our findings suggest that the multi-barcode DNA metabarcoding method assessed in this study can provide a composition of more diverse sets of single drugs and polyherbal formulations listed in the Ayurvedic Pharmacopoeia of India. We obtained 100% average detection efficiency and relative fidelity of targeted plants for single drugs and 79% for polyherbal formulations through the multi-barcode sequencing approach. We have primarily focused on detected plant species in herbal products rather than undetected plant species because many steps, such as DNA extraction biases, PCR biases, and manufacturing processes, that can lead to DNA degradation or loss beyond detectable limits, failing to detect plant species. The presence of non-targeted plant species in herbal products could be due to unintentional contamination of the supply chain, economically motivated adulteration, and/or admixture of other species. Our study showed that the *rbcL* metabarcode had better detection ability for certain plant species, e.g., *O. tenuiflorum, J. adhatoda*, and *A. paniculata*, while *ITS2* had better discrimination power for certain plant species, e.g., species of the genus *Terminalia*, *Asparagus, Piper*, *Phyllanthus*, and *Pedalium murex.* Thus, the complementary approach of both metabarcodes is a promising tool for quality evaluation of herbal products and pharmacovigilance. However, the development of standardized methods for metabarcoding sequencing and bioinformatics analysis pipeline and curated database is needed for effective use as a regulatory tool to authenticate herbal products in combination with advanced chemical methods to identify bioactive therapeutics.

## Data availability statement

The datasets presented in this study can be found in online repositories. The names of the repository/repositories and accession number(s) can be found in the article.

## Author contributions

TT: performed experiments, established metabarcoding data analysis pipeline, data analysis, writing and editing manuscript, and validation of final manuscript. AS: performed experiments, data analysis, writing and editing manuscript, and validation of final manuscript. RP: designed primers for metabarcoding, performed experiments, established metabarcoding data analysis pipeline, and manuscript editing. SS: performed experiments, and manuscript editing. CJ: project administration, methodology, supervision, and review & editing. MJ: principal investigator, conceptualization, methodology, supervision, and review & editing. All authors contributed to the article and approved the submitted version.
